# Estrogens and the risk of breast cancer: A narrative review of literature

**DOI:** 10.1016/j.heliyon.2023.e20224

**Published:** 2023-09-17

**Authors:** Khayry Al-Shami, Sajeda Awadi, Almu'atasim Khamees, Ahmad Malek Alsheikh, Sumaiya Al-Sharif, Raneem Ala’ Bereshy, Sharaf F. Al-Eitan, Sajedah H. Banikhaled, Ahmad R. Al-Qudimat, Raed M. Al-Zoubi, Mazhar Salim Al Zoubi

**Affiliations:** a Faculty of Medicine, Yarmouk University, P.O Box 566, 21163, Irbid, Jordan; b Department of General Surgery, King Hussein Cancer Center, Amman, 11941, Jordan; c Department of Public Health, College of Health Sciences, QU-Health, Qatar University, Doha, 2713, Qatar; d Surgical Research Section, Department of Surgery, Hamad Medical Corporation, Doha, Qatar; e Department of Biomedical Sciences, College of Health Sciences, QU-Health, Qatar University, Doha, 2713, Qatar; f Department of Chemistry, Jordan University of Science and Technology, P.O.Box 3030, Irbid, 22110, Jordan

**Keywords:** Estrogen, Oestrogen, Risk, Breast cancer, ER

## Abstract

In female mammals, the development and regulation of the reproductive system and non-reproductive system are significantly influenced by estrogens (oestrogens). In addition, lipid metabolism is another physiological role of estrogens. Estrogens act through different types of receptors to introduce signals to the target cell by affecting many estrogen response elements. Breast cancer is considered mostly a hormone-dependent disease. Approximately 70% of breast cancers express progesterone receptors and/or estrogen receptors, and they are a good marker for cancer prognosis. This review will discuss estrogen metabolism and the interaction of estrogen metabolites with breast cancer. The carcinogenic role of estrogen is discussed in light of both conventional and atypical cancers susceptible to hormones, such as prostate, endometrial, and lung cancer, as we examine how estrogen contributes to the formation and activation of breast cancer. In addition, this review will discuss other factors that can be associated with estrogen-driven breast cancer.

## 1 Introduction

**Fig. 1:**
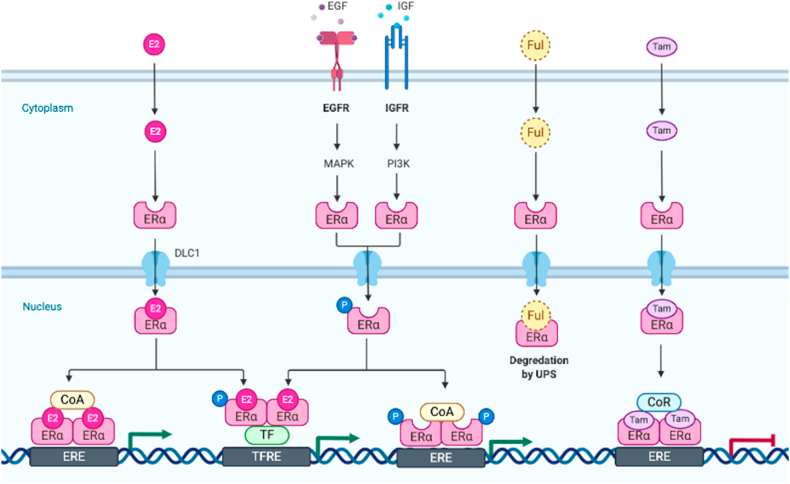
Signaling Pathway of Estrogen Receptor. Estrogen can cross the plasma membrane, where it interacts with intracellular ER and ER to affect DNA directly. The GPER1 and/or ER and ER can interact with estrogen to cause it, to activate intracellular signaling cascades as an alternative. Estrogen-mediated signaling events can be classified as genomic and non-genomic due to differences in the cellular and molecular processes regulating gene expression, in which estrogen-receptor complexes can bind to DNA directly or indirectly. The migration of estrogen-receptor complexes into the cell nucleus and direct contact with chromatin at particular DNA sequences known as estrogen response elements are two examples of genomic impacts (EREs). More than one-third of human genes controlled by estrogen receptors are reported to lack ERE sequence elements, although EREs have been found in multiple gene promoters and regulatory regions. Contrarily, non-genomic impacts entail the indirect control of gene expression via a range of intracellular signaling occasions. Below is a description of the known ways through which estrogens regulate both genomic and non-genomic aspects of gene expression.

Estrogens also known as oestrogens, are key hormones responsible for the progression and regulation of mammal females’ reproductive system and have an essential role in the non-reproductive system [1,2]. In addition, they are pleiotropic steroids that play a regulatory role in a myriad of physiological processes from reproduction to lipid metabolism [3]. Estrogens perform their action through two different types of receptors: First, classical nuclear estrogen receptors (ER) with two various isoforms known as (ERα and ERβ) that are encoded by genes on chromosomes 6 and 14, respectively. Second, novel cell surface membrane receptors (GPR30 and ER-X). Both kinds of estrogen receptors are expressed in the brain and periphery with cell and tissue-specific circulations [4,5]. Estrogen receptors are a component of the superfamily of nuclear transcription factors with a classical pathway of estrogen-dependent function. The action of estrogen receptors achieves in the cytoplasm by binding lipophilic hormone molecules and transferring the compound to the nucleus, dimerization, and interaction with suitable response elements in gene promoters region, which initiates transcription after co-activators binding as demonstrated in Fig. 1 [6,7]. Due to the significant role of ER in signaling transduction, the current review aimed to summarize the mechanism of action of estrogen in cancer development, particularly in breast cancer.

BioRender (2021). Estrogen Receptor Signaling. Retrieved from https://app.biorender.com/biorender-templates/t-5db2fd9f9688420082acf5d3-estrogen-receptor-signaling.

## 2 Estrogen synthesis

**Fig. 2:**
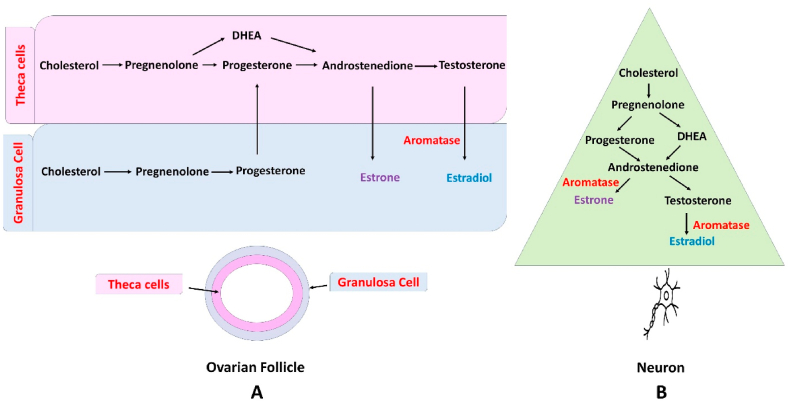
Estrogen synthesis pathway in the ovary and brain. (A) Synthesis of estrogens begins with the production of pregnenolone from cholesterol, catalyzed by the cytochrome P450 side-chain cleavage enzyme (P450scc). The pregnenolone is transformed into progesterone by 3 b-hydroxysteroid dehydrogenase (3 b-HSD) in both thecal and granulosa cells. Progesterone is transformed to androgens by cytochrome P450 17a-hydroxylase (P45017a) and 17 b-hydroxysteroid dehydrogenase (17 b-HSD) in the thecal cells throughout the follicular phase. The transformation of E2 is enhanced by the aromatase enzyme (P450Arom) in granulosa cells. (B) Neurons express all of the mandatory enzymes for the production of estrogen to create brain estrogen [12].

**Fig. 3:**
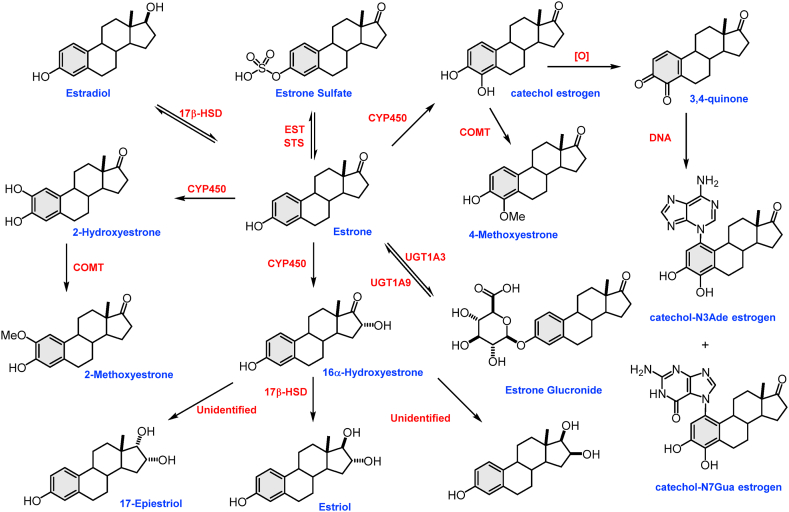
Estrogen metabolism pathway in humans. The diagram shows the metabolism of estradiol and other natural estrogens such as estrone and estriol. It demonstrates that conjugation (e.g., sulfation and glucuronidation) occurs in the case of estradiol and metabolites of estradiol that have one or more available hydroxyl (–OH) groups. Catechol and quinone formation from estrone is shown and how the derivatives are reacting with DNA to form depurination DNA adducts (adapted from Ref. [16]).

In premenopausal women, estrogens are synthesized primarily in theca cells in the ovaries, placenta, and corpus luteum. A noteworthy quantity of estrogens can also be created by non-gonad organs, like the liver, skin, brain, and heart. The synthesis process ends with the conversion of androgens to estrogens in granulosa cells by the aromatase enzyme as illustrated in Fig. 2A and B [8]. There are three main endogenous forms of physiological estrogens in women: estrone (E1), estradiol (E2), and estriol (E3). After menopause, E1 has an important role as it is formed in adipose tissue from adrenal dehydroepiandrosterone. While E2 which is also called estradiol, considers the major and most potent product of the estrogen biosynthesis process. E3 form is the least prevalent estrogen and is formed from the E1 or E2 (Fig. 3). Additionally, it plays a larger role during pregnancy when it is produced in large quantities by the placenta [[9], [10], [11]].

## 3 Estrogen metabolism in humans

Estrogens are metabolized via hydroxylation and conjugation. Hydroxylation is achieved by cytochrome P450 enzymes such as CYP1A1 and CYP3A4, while conjugation is performed by estrogen sulfotransferases (sulfation) and UDP-glucuronyltransferases (glucuronidation). Moreover, estradiol is dehydrogenated by 17β-hydroxysteroid dehydrogenase into the less common estrogen estrone as shown in Fig. 3. These reactions take place primarily in the liver, but also in other tissues [[13], [14], [15]].

### 3.1 2-Hydroxylation pathway

2-hydroxylation is the main hydroxylation pathway. CYP1A1 and CYP1B1; cytochrome P-450 enzymes which are expressed in breast and liver tissues are major phase I enzymes [17]. C2 hydroxylation for parent estrogens to catechol estrogens is catalyzed by cytochrome P-450 enzymes including CYP1A2 [18].2-Hydroxylated estrogens have a low binding affinity for the estrogen receptor [19]. Compared with estradiol these metabolites reduce hormonal potency and are the cause of non-estrogenic and anti-estrogenic activities. Some studies showed that 2-hydroxyestrone and 2-hydroxyestradiol can inhibit cell growth and proliferation [20]. Also, they have a role in normal cell differentiation and apoptosis [21]. Therefore, some researchers define 2-hydroxyestrone as a “good estrogen” [22]. The low potency or non-tumorigenic effect of the 2-hydroxy metabolites can be attributed to the high clearance rate, O-methylation by the COMT enzyme, which may inhibit tumor cell proliferation and angiogenesis [19]. In addition, it has been demonstrated that when COMT is blocked or when 2-hydroxyestrogens undergo redox cycling, they can cause DNA damage and release free radicals [23]. Moreover, some studies showed that methoxyestrogens like 2-methoxyestradiol inhibit carcinogenesis through microtubule destabilization [24,25].

### 3.2 4-Hydroxylation pathway

In liver microsomes, the 4 hydroxylations of estradiol primary start with the CYP3A4/3A5 enzyme [26]. 4-Hydroxylated catechol estrogens showed a carcinogenic potential because of their ability to damage the DNA by depurination adducts and oxidative damage that may initiate breast cancer [16,27]. As a biochemical marker, the ratio of 4-/2-hydroxyestradiol is used to differentiate malignant breast tumors because the production of 4-hydroxyestradiol is four times higher than 2-hydroxyestradiol in adenocarcinoma [28]. Additionally, it has been demonstrated that, as compared to control women, women with breast cancer or at high risk of developing breast cancer had much greater ratios of quinone-estrogen DNA adducts to their parent or conjugated catechol estrogens [29]. Moreover, some studies showed that the 4-methoxyestrogens prevent the oxidative metabolism of estradiol and oxidative DNA damage [30]. Although other studies showed that inhibition of the COMT enzyme was linked with higher levels of depurination 4-hydroxyestrone linked with higher levels of depurination 4-OH Estrone/Estradiol-DNA adducts [31].

### 3.3 16-Hydroxylation pathway

In the 16-hydroxylation pathway, 16α-hydroxyestrone is the main product. 16α-hydroxyestrone showed a potential tumor stimulation by catalyzing unprogrammed DNA synthesis and promoting independent growth in mammary epithelial cells [32,33]. Some animal studies showed that urinary concentration of 16α-hydroxyestrone is accompanied by an elevated proliferation of mammary cells and mammary tumor incidence [34,35]. Another study shows that there is a relation between estradiol 16α-hydroxylation and increasing the risk of developing breast cancer in humans, the levels of 16α-hydroxyestrone were eight times higher in the cancerous units of mammary terminal duct lobular in comparison with the nearby mammary fat tissue, which suggests a critical role of 16α-hydroxyestrone production in breast cancer induction [36].

## 4 Biological function of estrogen in the human body

Estrogens are found in both males and females; they are usually present at higher levels in women during reproductive age. They control the improvement of women's secondary sexual characteristics, such as breasts, and are elaborated in the thickening of the endometrium and other features of regulating the menstrual cycle. In men, estrogen regulates specific functions of the reproductive system essential to sperm maturation [8,37]. Estrogen receptors are responsible for mediating estrogen actions and functions; a dimeric nuclear protein binds to DNA and has a role in controlling gene expression. Similar to the principle of the other steroid hormones, estrogen moves in passively into the cell and binds to it then activates the ER [38].

## 5 Estrogen receptor (positive and negative)

ER-positive tumors overexpress the ER while tumors that contain a small number of receptors and sometimes no receptors are called ER-negative which is directing the treatment options [39]. Patients with ER-negative have lower survival rates in the first few years and their tumors are usually more aggressive [40,41]. However, 10 years after the initial diagnosis of the tumor without being associated with other health problems, the possibility of relapse is more in patients who have ER-positive [42]. In addition, other factors affect the life of a breast cancer patient, such as the infiltration of lymphocytes, especially for patients who have the disease before the age of forty, as the presence of a large number of CD8^+^ T lymphocytes contributes to the high survival rates of the patient, and this shows It is more clearly in patients who have ER-negative compared with patients with ER-positive [43].

## 6 Estrogen and estrogen receptors role in cancer development

The α and β isoforms of estrogen receptors exhibit similar structural and functional organization [3]. Both receptors interact in the same way with endogenous estrogens, mostly with 17β-estradiol (E2) [44]. E2 plays an essential role in the development and malignant progression of multiple cancers. The oncogenic function of estrogens is considered in both classical and non-classical hormone-sensitive carcinomas such as prostate, breast, endometrial, lung, colon, and ovarian cancers [[45], [46], [47], [48]]. The molecular basis of cancer initiation by estrogen has been suggested through the production of aromatic estrogen metabolites (catechol estrogens quinones) that are derived from normally formed catechol estrogens. Chemically, depurinating DNA-adducts are formed by the reaction of 4-OHE_1/2_ or 2-OHE_1/2_ with Adenine/Guanine bases which leads to DNA mutations (Fig. 3) [16].

### 6.1 Endometrial cancer

Tumoral ER expression is mentioned in approximately 30 different kinds of cancer, predominately in hormone-sensitive tumors like ovarian, breast, prostate, and endometrial cancers [49,50]. By utilizing immunohistochemistry (IHC), studies were able to compare ER protein expression with clinicopathological characteristics in tumor tissue and illustrated differential relations to the prognosis of disease based on the localization of cells and cancer type [48]. Endometrial cancer which is considered the most popular type of uterine cancer, using histopathology, can be subdivided into two types [51]. Type I endometrial tumors, also called low-grade endometrioid, form most of the endometrial cancer cases around 85%, usually express high levels of α estrogen receptor (αER), and are supposed to be hormonally driven [52]. Type II tumors contain high-grade endometrioid tumors, clear-cell, serous tumors, carcinosarcomas, and tumors with diverse histology. These tumors are expressing ER at low levels, have a worse prognosis, and have combined molecular features with serous ovarian cancer and triple-negative breast cancer such as a high prevalence of p53 mutations and a high number of copies of a variation [[53], [54], [55]].

### 6.2 Ovarian cancer

Extensive research in epidemiology has illustrated that hormonal and reproductive exposures are linked with a high risk of ovarian cancer. However, how these factors impact ovarian carcinogenesis and lead to tumor development is still not fully understood. Epithelial ovarian cancers are heterogeneous in their morphology, gene and protein expression [[56], [57], [58]]. These variations are mandatory to understand the etiology, prognosis, and treatment of ovarian cancer [59].

Studies have demonstrated differential associations between the risk factors of ovarian cancer and α-ER and progesterone receptor (PR) status, while no previous research has observed associations of ovarian cancer risk factors with β-ER expression [60,61]. Some reports suggested tumor suppression activity of ERβ in ovarian tissues and showed low expression in malignant transformation; with cell localization pattern [[62], [63], [64], [65]]. A study done by De Stefano and colleagues to distinguish between normal and cancer ovarian cells found that; ERβ staining was more likely to be localized in the nucleus in normal ovarian tissue while it was more likely to be localized in the cytoplasm in ovarian cancer cells [66]. Additionally, higher levels of expression of the ERβ protein have been linked with enhanced progression-free survival, furthermore, the probability of metastasis of lymph nodes has declined in serous tumors [67,68]. A recent study showed alterations in expression and localization of ERβ noticed to be essential for causing ovarian cancer [59].

### 6.3 Prostate cancer

It is already known that the estrogen receptor signaling pathway is biologically relevant in prostate cancer but has been relatively under research as a biomarker in prostate tumors. Estrogen has a significant part in physiological hormone signaling, and circulating levels can be distinguished in males [69]. Presently, it is not fully known if direct signaling through ER has an impact on prostate cancer behavior in models or patients. Estrogen-based treatments were one of the first effective therapy choices for advanced prostate cancer and are still utilized in many countries [70]. Its therapeutic effects were thought to be the effect of chemical castration which results from negative feedback on the hypothalamic-pituitary-testicular axis [71,72]. By utilizing knockout mice lacking the aromatase enzyme in previous studies, the absence of the production of estrogen prevented prostate cancer progression despite the raised levels of testosterone [73].

## 7 Estrogen and estrogen receptors and risk of breast cancer

### 7.1 Estrogen role in breast development

Breast cancer is the m ost common female cancer with no decline in the incidence, prevalence, or mortality. In 2023, it has been estimated that about 300 000 new cases of BC will appear with more than 43 000 deaths in the USA [74]. In 2020, breast cancer surpassed lung cancer to become the most frequently diagnosed cancer and ranked fifth among the leading causes of cancer-related fatalities worldwide. During that year, there were approximately 2.3 million reported cases and 685 000 documented deaths attributed to breast cancer [75]. In combination with growth hormone (GH) and its secretory product insulin-like growth factor 1 (IGF-1), estrogen is important in mediating breast development during puberty and breast maturation during pregnancy to prepare for lactation [76,77]. Estrogen is highly involved in breast development and it is primarily responsible for making the ductal component of the breast as well as for causing growth in fat deposition and connective tissue [[78], [79], [80]]. Moreover, it is indirectly elaborated in the lobuloalveolar component, via increasing the expression of progesterone receptors in the breasts and by inducing prolactin secretion. After working with estrogen, progesterone, and prolactin together, they can complete the growth of the lobuloalveolar during pregnancy [78,81,82].

### 7.2 Expression and distribution of ER in the breast

The expression of the individual isoforms of ER is regulated differently in the breast epithelium, compared with other tissues [83]. ERα and ERβ show some distinct expression patterns; ERα is controlled in the luminal epithelial compartment, while ERβ is expressed in myoepithelial cells and luminal, as well as the endothelium of blood vessels and stromal cells [84]. Interestingly, these isoform-specific expression manners can differ between species, for instance, in the rat mammary gland, the ERβ is expressed throughout all stages of development, while ERα demonstrates fluctuating expression, it increases during puberty and declines during pregnancy, as well as increases during lactation, and decreases again in the post-lactating gland [85]. On the other hand, in the rhesus monkey, neither ERα nor ERβ could be noticed in the lactating mammary gland, nor was PR detected, this is a confirmation that observations that are noticed in animal models, may not always be prolonged into the human breast [86]. Therefore, the expression of steroid hormone receptors in normal breast tissue is highly dependent on cell type, the stage of progression, and the exposure to cycling endogenous or exogenous hormone utilization.

### 7.3 Estrogen and breast cancer

The ovarian hormones of females, estrogen, and progesterone are essential regulators in the development and function of normal breasts, as well as critical in breast cancer. The breast is developmentally infrequent in the fact that the main part of the improvement of the breast happens postnatally, during puberty, and at the onset of pregnancy [87]. Both estrogen and progesterone are censoriously involved in these normal evolving processes, having highly coordinated functions in the development of the ductal structures and amplification of lobules of the normal epithelium. It looks that these behaviors become undermined in the development of breast cancer, connecting both steroids in the enhancement and progression of cancer [83].

**Fig. 4:**
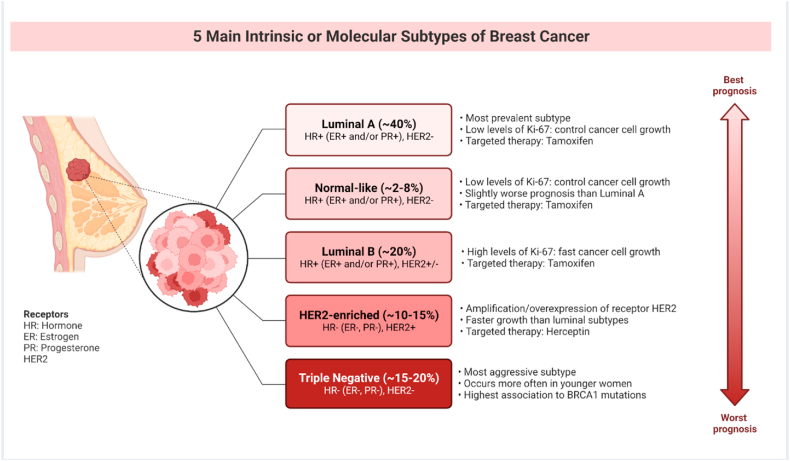
The five main subtypes of breast cancer; are where the best prognosis is when the ER is positive, while the worst is a triple-negative case when ER is negative. BioRender (2021). Intrinsic and Molecular Subtypes of Breast Cancer. Retrieved from https://app.biorender.com/biorender-templates/t-5f872409fb2c3900a82e109e-intrinsic-and-molecular-subtypes-of-breast-cancer.

Breast cancer is considered mostly a hormone-dependent disease [88,89]. Approximately 70–80% of breast cancers express progesterone receptors and/or estrogen receptors, and if they are found in a tumor, are a good mark as a promising prognostic biomarker [89,90]. In addition, their expression in malignant cells is mostly associated with other tumor characteristics. The positive association between PR, ER, and prognosis has been identified with the progress of multi-gene prognostic processes that categorize breast cancers into clinically relevant groups, with PR and ER segregating into the better distinguished luminal cancer subtypes as demonstrated in Fig. 4 [91,92]. For managing ER + breast cancer, using agents targeting the signaling pathway of estrogen is still the most effective treatment [83].

An abundance of clinical and experimental data in various studies has illustrated that estrogen is critical in the progression and proliferation of breast cancer [93,94]. In different malignant breast cells, the function of an ER signaling pathway is to promote unequal rates of cell proliferation and apoptosis, with pro-survival and proliferation signals devastating pro-death and quiescence signals [95]. Dependable on a pro-proliferative role, there is in vitro proof that estrogen can inhibit apoptosis in breast cancer cells by up-regulation of Bcl-2, an anti-apoptotic proto-oncogene. As a result of its part in the proliferation and growth of tumors in breast cancer cells, the ER signaling network has been considered an attractive agent for the development of therapeutic targets [96].

## 8 Factors affecting breast cancer

### 8.1 Obstetric and gynecological factors

#### 8.1.1 Menstrual cycle

The menstrual cycle is a physiological process that starts in females at ages ranging from 8.5 to 13 years old, and it repeats in a cycle-like pattern that varies in length from 25 to 34 days until they finally stop experiencing it for one year entering a stage called menopause at an average age of 51 years old [97]. The cycle alternates between two phases as it happens each time, a follicular phase that ranges from 10 to 16 days, and a luteal phase that is usually the same in all women that is 14 days. This makes the follicular part of the cycle the usual reason for its length difference between women, what mainly regulates this process are hormones secreted from the hypothalamus, the pituitary gland, and the ovaries like estrogen and progesterone [98], which induce different changes in the reproductive organs leading to the capability of fertility [99]. Accordingly, there is variability in the menstrual cycle's age of onset, length, and age of termination between different individuals, but does that have any role in increasing the risk of developing breast cancer in women? And does estrogen play a role in that risk?

Understanding the relationship between the menstrual cycle and breast cancer could be beneficial in diagnosing and treating breast cancer since the aggression and poor prognosis characteristics of breast cancer in premenopausal women could be due to the fact of the influence of menstrual cycles on estrogen receptor-positive subtypes of breast cancer, which are the most common in that age group.

##### 8.1.1.1 Age of menarche and menopause

The age of menarche and time of menopause seem to affect the risk of developing breast cancer, due to their role in the time and amount of exposure to hormones like estrogen and progesterone. An earlier age of menarche induces a rise in breast cancer risk since it causes earlier exposure to the hormonal changes that induce the beginning of menstrual cycles. As well as, a rise in estrogen levels in the first couple of years following early menarche that can remain throughout their fertile years [100]. This risk is limited to hormone receptor-positive subtypes of breast cancer [101]. Moreover, the same concept of increased estrogen exposure is related to the increase of breast cancer risk related to a late age of menopause, due to more exposure to menstrual cycles and their hormones, although the increase in that risk may be not evident up until 10–20 years after menopause [100].

##### 8.1.1.2 Menstrual cycle length and regularity

The regularity and length of the menstrual cycle both have an association with breast cancer risk, a short length, and a regular cycle are associated with breast cancer occurrence since more menstrual cycles result in a shorter follicular phase and therefore more dominant exposure to progesterone and an increased division of epithelial cells in the fixed luteal phase each cycle [102]. Despite some data suggesting no relation between menstrual cycle irregularity and breast cancer with an exception of a precise group of women aged 30–34 years old with a high level of irregularity [103]. Likewise, no relation between irregularity and increased length of menstrual cycles and breast cancer risk again with the exception of longer menstrual cycle length during the age of 18–20 years old decreasing the risk of breast cancer in women younger than 40 years old [104].

#### 8.1.2 Pregnancy

Breast cancer that is diagnosed during pregnancy or after it the post-partum period has a poor degree of prognosis [105,106]. Many factors related to pregnancy are thought to influence breast cancer risk. The first one is the age of the woman when she had her first pregnancy, or “age at first pregnancy”. In general, women who first conceive at an older age have a higher risk of developing breast cancer, while younger women have a decreased risk of developing breast cancer. The mechanism behind that is that older women will go through more menstrual cycles and therefore be more exposed to substances like estrogen that can promote the carcinogenesis of breast tissue. Other than that, some changes occur in pregnancy that protective against breast cancer, like the hormonal changes that happen in early pregnancies that secure a gene called p53, which helps in the cessation of the cell cycle, therefore, stopping cell growth, another hormone change is the production of human chorionic gonadotropin, a hormone that differentiates breast cells and makes them less prone to a response by carcinogens [107]. However, age at first pregnancy doesn't affect all breast cancer subtypes in the same way, for example, triple negative and human epidermal growth factor receptor 2 (HER2) subtypes of breast cancer are not affected by it, while the luminal subtype of breast cancer has more prevalence among women who are older than 24 years old when they have their first birth [108]. The second factor is the number of pregnancies that the woman had regardless of whether it resulted in an abortion, stillbirth, successful childbirth, or “parity”. Parity has a protective effect against breast cancer, this could be due to the hormonal changes occurring during multiple pregnancies like decreasing estrogen levels and increasing progesterone levels, as well as increasing the differentiation of mammary tissue while decreasing the activation of its stem cells [109]. Nevertheless, the effect on breast cancer varies according to subtype, as it decreases the risk of developing hormone-receptor-positive subtypes of breast cancer mainly the HR+ and Ki-67 subtypes [110]. The time length between each birth can also play a role in breast cancer risk. Although shorter intervals between each childbirth (excluding stillbirths and abortions) showed a protective effect against the lobular breast cancer subtype being effective up to the fifth childbirth, a longer interval between the first and second childbirth provides protection specifically in premenopausal women against the ductal subtype of breast cancer [111].

The hormone levels during pregnancy, the length of the pregnancy, and the gender of the fetus have been suggested to be related to the development of BC. The hormone levels that decrease breast cancer risk when elevated are the human chorionic gonadotropin hormone (beta-HCG) decreasing it by 30% and the Alpha-fetoprotein hormone (AFP) decreasing it by 50%, while the hormone that increases breast cancer risk while elevated is the estrone hormone increasing it by 2.5 times [112]. Secondly, full-term pregnancy is associated with a lower risk of breast cancer in the long term but a higher one immediately following the birth after the full-term pregnancy is compensated with breastfeeding [113]. Lastly, the sex of the fetus has no relation to the breast cancer risk [114], however, in certain cases like hypertension induced in pregnancy also called “preeclampsia” a male fetus has an impactful decrease in the risk of developing breast cancer for the mother [115].

#### 8.1.3 Breastfeeding

Previous studies showed that breastfeeding per se has an overall reduction in breast cancer risk [116] with more impact on young women [117]. It may have different influences on the development of different subtypes of breast cancer. A systematic review, and meta-analysis by Lambertini M, Santoro L, Del Mastro L et al., reported a protective effect of breastfeeding against the chance of developing the luminal and triple-negative subtypes of breast cancer, whereas it does not affect the development of the HER2 subtype of breast cancer [108]. However, its effect on positive receptor subtypes of breast cancer is still unclear and needs more data to be determined. Breastfeeding can also have different effects depending on different BRCA gene mutation carriers, having a protective effect against breast cancer in the BRCA1 gene mutation carriers and no effect on the BRCA2 gene mutation carriers [118].

Studies demonstrated that longer breastfeeding duration and protects against breast cancer [119] [120]. For instance, breast cancer risk reduction by 26% and 37% if the duration exceeds a year [121]. Although this protection may only be limited only to postmenopausal women [122]. The mechanism behind this protection that the longer duration of breastfeeding provides is due to the breast cells differentiating after pregnancy to be able to lactate which decreases its responsiveness to substances like estrogen that can stimulate breast cells to become cancerous. Another way is the mechanical flushing of carcinogens and exfoliated DNA-damaged cells and insulin through breast milk, which reduces insulin levels in the blood and prevents the anti-apoptosis effects that insulin can have by increasing the level of substances like insulin-like growth factor [123]. Nevertheless, a recent review proposed there was still an unclarity of the definitive relationship between breastfeeding and breast cancer risk [124].

#### 8.1.4 Polycystic ovary syndrome (PCOS)

Polycystic ovarian syndrome (PCOS) is the most common metabolic disease occurring in women at the age of reproduction. Women with the condition are more prone to cardiovascular manifestations as well as insulin resistance, but it also affects fertility, hormone balance, and ovulation [125]. PCOS doesn't have a clear association with an increased breast cancer risk [[126], [127], [128], [129]], despite it causing changes in the body that can lead to breast cancer like the high androgen levels in the blood that results in the absence of ovulation and therefore longer exposure to estrogen [130]. The high levels of insulin that it causes in the blood, as well as having an intersection with a gene that is also present in breast cancer [131]. Also, it produces high levels of *anti*-Mullerian hormone (AMH) which could suggest PCOS is the original factor responsible for the increased breast cancer risk occurring in women with high levels of (AMH), not the increase in the hormone itself [131,132]. There are also some data like a population-based case-control study by Kim J, Mersereau JE, Khankari N et al., that does suggest it has a positive relationship with an increased risk of breast cancer occurrence specifically in premenopausal women [133].

### 8.2 Ethnicity and diet

Breast cancer is the most common malignancy among women regardless of their ethnic groups with a considerable difference in the incidence between populations [134]. For instance, the breast cancer risk among Asian American women is lower than it is in Caucasian American women with a small exception related to age and state [135]. This difference could be due to differences in lifestyles between them, like the diet rich in soybean content [136]. Another diet that has contents of foods lowering breast cancer incidence is the Mediterranean diet as opposed to a Western diet style, which increases that risk [137]. Other examples of foods associated with a higher risk of breast cancer are foods like Ultra-processed foods [138], red meats, higher than 450 g of milk intake per day [139], saturated fats [140], and alcohol [141,142]. However, fruits, vegetables, fatty fish [143], soybean [144], food containing β-carotenoids [145], mushrooms [146], and olive oil [147] are associated with lowering breast cancer risk. Fruits and vegetables specifically reduce the risk of postmenopausal breast cancer as well as estrogen, progesterone positive, and negative breast cancers [148].

### 8.3 Obesity

Weight and body mass index (BMI) can affect breast cancer by the high amounts of aromatase enzymes due to the high content of adipose tissue [149]. It has different effects on different breast cancer subtypes, increasing the incidence of triple-negative breast cancer in premenopausal women and decreasing the incidence of the luminal A subtype of breast cancer [150], but has a weaker effect on increasing breast cancer in postmenopausal women [151].

### 8.4 Microbiota and breast cancer

Dysbiosis was found to make an impact on the effectiveness of chemotherapy drugs and prefer the environment of tumor development, suggesting an association between gut dysbiosis and the progression of cancers, autoimmune disorders of the gut, or inflammatory diseases. Even with the presence of some skeptical concerns about whether breast cancer development is due to this dysbiosis or the natural selection of microorganisms that can survive in a carcinogenic environment with special nutritional requirements [152], a diversity of lipid types suggest lipid signatures for the bacterial growth species in breast cancer compared to healthy breast tissue [153]. Breast cancer environment was reached in higher numbers of Phylum *Proteobacteria*, families *Micrococcaceae, Caulobacteraceae, Rhodobacteraceae, Nocordioidaceae*, and *Methylobacteriaceae,* and genus *Propionicimonas* compared to benign healthy breast tissue, even with different types of breast cancer like HER2, Luminal A, Luminal B, ER+ the type of microbiome was diverse and different [154]. A shifting of microorganism types was estimated from the healthy breast tissue and cancerous including the presence of microorganisms that will raise the local breast estrogen exposure level by glucuronidation like *S*. *pyogenes* [155]. To support the role of breast microbiota in breast cancer either as an inducer or consequence of the disease, a study has been done at St. Joseph's Hospital in London, Ontario, Canada by collecting samples from women, aged between 19 and 90 with healthy breasts or with breast cancer. These women underwent breast surgery and the researchers found that breast cancer microbiota was made up of collocation of bacteria that end up with DNA damage and breaks in Vitro including *Bacillus, Enterobacteriaceae, Staphylococcus*, and *Escherichia coli* (a member of the Enterobacteriaceae family) and *Staphylococcus epidermidis* rising [156]. Fernández et al. support the relationship between the gut microbiota and breast cancer development in many ways. The gut contains many glucuronidase bacteria, including the Clostridium leptum cluster and the Clostridium coccoides cluster, which are members of the Firmicutes phylum. The first method, which included deconjugation of contacted estrogen, came from an endogenous source or even an exogenous source of estrogen through the bile pathway. This deconjugation process will be aided by the Proteobacteria phylum's Escherichia/Shigella bacterial group, which increases the blood level of estrogen. Additionally, the second method involves the function of Firmicutes and Bacteroidetes bacteria of the gut, which are in charge of processing the colon. These bacteria discovered that nutrients were present in much higher ratios among obese women, knowing that obesity is an indirect link between breast cancer and the gut microbiome [157]. The gut microbiome, which is thought to play a role in the development of breast cancer, should be the first thing to be targeted during breast cancer treatment or even prevention because there is a clear indication that there is a difference between microbial patterns in healthy breast tissue and women with breast cancer where many DNA breakers are found. Additionally, researchers should discover a means of preventing dysbiosis so that the microbiome balance in breast tissue remains constant and does not change [158,159]. The favorable effects of probiotic therapy on the treatment of breast cancer contradict the idea of the negative effects of microbiota and its function in breast cancer given that this bacterium also contributes to the metabolism of cytotoxic medicines [160]. Another study confirmed the beneficial effects of probiotics in preventing the growth and even genesis of breast cancer. By avoiding dysbiosis, we can improve the balance of the gut's metabolic activity and lower obesity, which is known to increase the risk of breast cancer [161]. Many bacteria found in breast cancer patients have an inverse relationship to the prognosis of breast cancer, a point that should be taken into consideration in the future of breast cancer treatment strategies. These bacteria may affect body weight, chemotherapy agents, and even the potential of neurological side effects [162].

### 8.5 Smoking

Breast cancer in active or passive smokers can't be negligible knowing that this tobacco smoke included a lot of carcinogens of breast tissue, especially mammary cells. The enzyme N-acetyltransferase 2 (NAT2) was found to play a role in the detoxification and eradication of tobacco smoke chemicals so genetic polymorphisms of this enzyme gene will play a role to determine whether you are a fast or slow acetylators. Moreover, being a slow acetylators with a long history of smoking increases the risk for breast cancer development two times. The onset of smoking was found to increase the risk, like young age or long history of smoking before the first term of pregnancy [163]. Jones et al. reported in their cohort study that the risk of breast cancer and smoking was potentially supported, especially if the onset of smoking was even before the menarche in young girls with a family history of breast cancer [164]. Breast cancer at early stages before lymph node metastasis or organ spread was found to begin at higher rates among smokers of younger age [165]. Another study found that smoking plays a significant role in the prognosis of breast cancer with higher rates of mortality before or after the diagnosis of breast cancer [166].

### 8.6 Oral contraceptive

Several reviews support the idea that oral contraceptives increase BC risk especially, in up five years of use or before the first pregnancy [167,168]. A similar study suggests that from first pregnancy and up the breast becomes well-differentiated and the carcinogenic effect is negligible of oral contraceptives on breast tissue especially mammary cells of the breast, compared to the established risk of oral contraceptives and breast cancer at the age before menarche even during the prenatal period [169]. The data is still not enough to powerfully support the relationship between the oral contraceptive in breast cancer and the variable impact of oral contraceptives on different breast tissue receptors, which are the main players in breast cancer development like estrogen receptor (ER), progesterone receptor (PR), human epidermal growth factor receptor 2 (HER2) [170]. A Cohort study found that women with positive BRCA1 and BRCA2 mutations developed a significant risk of breast cancer but only in the short term, while lifelong combined oral contraceptives (estrogen and progesterone) reduced the cancer risk, but some drugs like hormonal replacement therapy were found to interfere with this protective long term positive impact of this combined oral contraceptive [171]. Many mechanisms like the disruption of endocrine system balance or provoking breast cancerous cells or even promoting metastasis for present cancerous cells in the tissue of the breast, was found to be strongly related to the onset of the use of oral contraceptive despite the other relations like the combination with progesterone or long-term effect of oral contraceptive drugs [172]. Ovarian and endometrial cancer in oral contraceptive women users was found to protect against them compared with limited risk for breast cancer development [173].

### 8.7 Tamoxifen and aromatase inhibitors

Most women with breast cancer are in a premenopausal state and a wide range of patients are hormone receptor-positive. Therefore, the use of blockers for this hormonal receptor was the way of treatment for a long time like Tamoxifen, but in the last years with the appearance of aromatase inhibitors, the directions are more toward using aromatase inhibitors, especially with gonadotropin-releasing hormone (GnRH) agonists showed to have a promising effect on the treatment of hormone receptor-positive particularly in premenopausal women [174]. The adjuvant and neoadjuvant hormonal therapies with aromatase inhibitors with or without combination with tamoxifen showed different impacts on the effectiveness of the treatment and management of breast cancer. In neoadjuvant therapy, trials showed that aromatase inhibitors like (anastrozole, letrozole, and exemestane) are more effective than tamoxifen. Compared to conflicting outcomes in adjuvant therapy, the main component of which is tamoxifen. However, we can't deny the combination with aromatase inhibitors showed a powerful impact on breast cancer treatment despite the different regimens and duration of treatment in adjuvant therapy of breast cancer. Also, the risk of osteoporosis and thromboembolism should be kept in mind when dealing with the different toxic effects of each drug [175]. With the documented side effects of some breast cancer therapy even if they are not that common, we can overcome them with other medications like bisphosphonates for bone fracture with aromatase inhibitors. The use of aromatase inhibitors in premenopausal women with early-stage of estrogen receptor-positive (ER+) is not that effective compared to postmenopausal women to reduce the mortality rate, except if we use ovarian suppression agents by then the therapy may show the promising result in premenopausal women. So, in premenopausal women, tamoxifen will preserve its significant impact on mortality as indicated many years ago compared to aromatase inhibitors [176]. The recommendations from the American Society of Clinical Oncology (ASCO) panel to use an aromatase inhibitor in postmenopausal women especially the third generation of them including anastrozole, letrozole, and exemestane in estrogen-positive breast cancer with or without tumor metastasis i.e. as adjuvant therapy was more effective even before the tamoxifen therapy initiated [177]. In the reduction and even absence of estrogen source from ovaries in postmenopausal women, the adipose tissue represents the core fuel for estrogen receptor-positive breast cancer in postmenopausal women a process needed aromatase to catalyze the final and rate-limiting step in the biosynthesis of estrogen. Accordingly, using aromatase inhibitors in hormone receptor-positive breast cancer in postmenopausal women is still the gold standard treatment. Also, the availability of MEK inhibitors, Raf inhibitors, PI3K inhibitors, mTOR inhibitors, and Akt inhibitors support the guidelines to continue in the same rhythm of management of cancer in postmenopausal women despite the resistance issue of aromatase inhibitors by them [178]. Consequently, the resistance issue for estrogen-positive breast cancer that is treated with the hormonal therapy pathway is the main boundary against hormonal therapy. However, the addition of inhibitors for many of these resistant pathways along with hormonal therapy may represent the way of overcoming this challenge in estrogen-positive breast cancer treatment [179]. The treatment is not always the case, the directions even support the prevention and development of breast cancer by using hormonal agents like tamoxifen, especially in women with a family profile of breast cancer [180]. With the wide spread of nonsteroidal anti-inflammatory drugs (NSAIDs) finding a relation between using them and the risk or even prevention of breast cancer represent a matter of interest. A recent study published in 2020 reported that using nonsteroidal anti-inflammatory drugs (NSAIDs) after using Proton pump inhibitors showed to reduce the risk of breast cancer. This is a promising result that needs further investigations and studies to generalize the results [181]. Likewise, statins for a long time, used for controlling elevated cholesterol levels and maintenance of the prognosis of ischemic artery diseases, even approved using them as tumor-killing medication with an established role of them in decreasing the mortality and the recurrence of breast cancer have been found in the patients. On the other hand, poor data about using statins for targeted therapy for breast cancer particularly with local or metastasis conditions or even in the wide diversity of breast cancer types [182].

### 8.8 Local hormone therapy

One of the postmenopausal complications in women is the complaining of genital changes like vaginal atrophy and symptoms of hot flashes due to changes in the hormonal balance mainly of estrogen in their body at that period of their life. Accordingly, the availability of synthetic topical estragon analog to relieve genital symptoms only showed no link to increases in the risk of heart disease or cancer risk in general [183]. So the effectiveness of topical estrogen for the treatment of genital and even urological symptoms as well in postmenopausal women can't be denied, but we should be aware of the risk of recurrence of breast cancer secondary to estrogen medications for those purposes [184]. In diagnosed breast cancer patients who are on therapies like aromatase inhibitors, it showed its effect on the genitalia of the women ranging from simple dryness to petechial bleeding and painful intercourse. Therefore, using topical estrogen may relieve these symptoms, and even though it should be used with caution, the patient must understand that using this topical estrogen that will relieve the genital symptoms may increase the recurrence rate of breast cancer that she already had [185]. Nevertheless, insufficient statistical evidence was estimated for the risk of breast cancer recurrence for patients who were treated with aromatase inhibitors and using a topical hormonal treatment for vaginal symptoms like dryness at the same time. However, patients who were treated with tamoxifen showed no elevated risk of recurrence compared with aromatase inhibitor-treated patients [186]. Another study suggests that the postmenopausal women who were treated with aromatase inhibitors and took topical estrogen treatment for the vaginal symptoms secondary to aromatase inhibitors showed poor systematic uptake indicating an indirect to decrease the suspicion of using this topical hormonal therapy and breast cancer recurrence [187]. The alternatives like selective estrogen receptor modulators (SERM) e.g. ospemifene and others like promestriene are better compared to the risky treatment by vaginal estrogen at least for short-term therapy for vaginal symptoms [188].

### 8.9 Hormone replacement therapy

Hormonal replacement therapy showed effective outcomes in the treatment of menopausal symptoms in women like hot flashes [189]. A Nested case-control study concluded that breast cancer risk, especially lobular type, was increased by a long period of using hormonal replacement therapy [190]. Women with the famous breast cancer mutations BRCA1 and BRCA 2 who did prophylactic salpingo-oophorectomy (RRSO) followed the use of hormonal replacement therapy showed no effect on the breast cancer risk at least for estrogen formulation alone of hormonal replacement therapy [191]. Topical forms of hormonal replacement therapy or progesterone alone hormonal replacement therapy were reflected in opposite to estrogen or combined estrogen-progesterone hormonal therapy to not linked with elevating the risk of breast cancer. Other factors like body weight play a role in directing the risk, which shows slim women with higher risk compared to obese ones who used hormonal replacement therapy [192].

## 9 Prenatal estrogen exposure and the risk of breast cancer

The variety of estrogen concentrations during pregnancy among pregnant women is explained by the impact of numerous exogenous variables on estrogen levels in the intrauterine period. In comparison to estrogen during the intrauterine period, when levels are ten times higher, the adult lifetime estrogen level is often significantly lower. Furthermore, estrogen is one of the key players in the development of breast cancer. Trichopoulos put up a theory that claims that the risk of breast cancer starts even before conception, or in utero [193,194].

To calculate the chance of getting breast cancer later in life, many studies employed indirect indicators of estrogen levels during pregnancy. Some of these indicated factors, such as greater mother age and having twins, have been linked to an increased risk of breast cancer after pregnancy, while others, such as maternal smoking, have been linked to a decreased chance of developing breast cancer in later life [195]. Even the chance of acquiring breast cancer varies amongst twins, according to a study, with dizygotic twins exposed to more estrogen having a higher risk than monozygotic twins [196]. Diethylstilbestrol (DES), an exogenous estrogen analog, was used by women to stabilize pregnancies and lower the chance of several pregnancy problems, including abortion [197]. It was discovered to contribute to the later development of breast cancer in their daughters, which was explained by its influence on DNA methylation by raising DNA methyltransferases (DNMTs) levels, which in turn affected the genes of the mammary gland in the breast that are responsive to estrogen [198,199].

However, other research revealed that conditions including pre-preeclampsia and twins will secrete less potent forms of estrogen-like E3 and E4, and even in rare cases, may lower intrauterine estrogen [200]. For the first three months of pregnancy, when an increase in several hormone levels, including estrogen, may indicate that a protective factor has not been triggered, birth weight and its relationship to hormonal levels during pregnancy are not a particularly promising indicator for the risk of breast cancer [201]. Given that most left-handed people are at a lesser risk for breast cancer, an interesting study discovered that early life androgen, specifically testosterone in comparison to estrogens, is proposed as preventive against the disease [202]. The Digit ratio, which not only predicts the probability of getting breast cancer but also the onset of it, is another promising study of predicting breast cancer risk using non-invasive techniques. Which is how it depicts the prenatal testosterone level that may be linked to a lower risk of breast cancer [203].

## 10 Fibroadenoma

Fibroadenoma of the breast is common among all ages with a peak incidence during the second and third decades occurring in 25% of females [189,204]. Histologically, the fibroadenoma is a biphasic tumor consisting of an epithelial and a stromal component. The epithelial component of it is similar to normal breast epithelium [205]. However, the incidence of carcinoma in situ and invasive carcinoma originating from fibroadenoma is 0.3% [205]. Moreover, the classification of fibroadenomas is based on their histology and size. Simple fibroadenomas are the most common type and can occasionally manifest as a smooth movable mass up to 3 cm in diameter. Giant fibroadenomas are less common but can appear throughout adolescence [206].

The clinical assessment of fibroadenomas involves clinical examination (history, physical examination) imaging, and non-surgical tissue biopsy (triple test) [207]. It is usually present as an encapsulated, mobile, rubbery, non-tender mass [208]. Ultrasound is used in younger women while mammography is combined with ultrasound in older women for the diagnosis of fibroadenoma. Notwithstanding, the most accurate way to confirm the diagnosis is tissue biopsy, which is performed by either fine-needle aspiration or core biopsy [207]. Fibroadenomas are accounting for ≤50% of all breast biopsies in Tokyo, New York, and Nigeria [189]. Asymptomatic fibroadenomas are managed conservatively while symptomatic fibroadenomas are managed by surgical excision [207].

As mentioned earlier, the fibroadenoma is a biphasic breast lesion in which both epithelial and stromal components of the terminal ductal unit proliferate [205]. The primary event is usually thought to be stromal cell proliferation, followed by epithelial cell proliferation. Furthermore, as women age, the stroma becomes less cellular and more hyalinized [209]. Although these findings occur in young women and sclerotic involution in the elderly, the fibroadenoma is hormonally dependent [210]. A recent discovery of higher plasma levels of estradiol in patients with fibroadenoma supports this hypothesis [211].

The estrogen receptor (ER)-α is the traditional mediator of the response to estradiol. Although ER-α is mostly expressed by epithelial cells in fibroadenoma, its expression by stromal cells remains controversial. According to a recent investigation, only the ER-β isoforms were detected in the stromal cells of adult human mammary glands. Recent studies revealed that ER-β activates a heterogeneous fibroblast population with different lifespans in the stroma of fibroadenomas. In fibroadenomas, ER-β-related myofibroblastic differentiation of fibroblasts may influence matrix remodeling and inhibition of the sclerotic involution [211].

In young premenopausal women, the incidence and development of fibroadenoma are dependent on their reproductive history and the presence of ovarian hormones. In addition, environmental factors may affect endogenous estrogen levels and cause hormonal dysfunction, and menstrual fluctuations, which may increase the risk of fibroadenoma, and some lifestyle factors with antiestrogenic effects may reduce the risk of fibroadenoma. Also, early pregnancy appears to prepare the breast for lactation, while estrogens and other circulating hormones stimulate the fast proliferation of epithelial breast tissue, followed by hormonally driven mammary epithelium differentiation. This early differentiation could prevent epithelial cells from developing fibroadenoma, which is caused by estrogen-dependent hyperplasic processes. Even while the preventive effects of OCPs containing >50 mcg estrogen (but not for progestogen-only OCPs) were suggested, the current studies showed that the incidence of fibroadenoma is not higher in women who use oral contraceptives for longer periods. Likewise, severe stress, which can raise endogenous estrogen levels, was considered another possibility in this aspect. However, further research is needed to determine the involvement of psychological illnesses in the occurrence of fibroadenoma [212].

## 11 Fibrocystic breast disease (FBD)

### 11.1 Pathophysiology of FBD

Fibrocystic changes refer to several clinical and histological abnormalities in the female mammary gland. Some of these should be viewed as a disorder of physiological development, maturation, and involution rather than a disease. Moreover, fibrocystic changes are common in around 50% of all women over the age of 30 ^204^. This disease progresses with premenopausal age and is most noticeable in women in their forties. While fibrocystic changes regress during the postmenopausal period. The pathophysiology of fibrocystic changes is determined by estrogen dominance and progesterone deficiency, which results in connective tissue hyperproliferation and fibrosis and is followed by facultative epithelial proliferation. Likewise, the risk of breast cancer is increased two to fourfold in these patients [213]. Proliferative and non-proliferative fibrocystic changes are classified according to their risk [204].

One of the key pathophysiological issues in fibrocystic breast diseases is the prevalence of elevated estrogen concentration throughout the menstrual cycle [214]. Likewise, changes in the concentration of steroid receptors and their affinity for estradiol may cause hormonal imbalance, which leads to the interlobular connective tissue to accumulates mucosal edema under the influence of estrogen, resulting in swelling and hyalinization. All of these factors could have an impact on the appearance of mastopathy and sclero-cystic changes. Estrogen stimulates the synthesis of DNA, mitotic activity, differentiation, and proliferation of breast cells and connective tissue at the cellular level [215].

### 11.2 Clinical assessment of FBD

The clinical assessment of fibrocystic changes involves clinical examination (history, physical examination) imaging, and fine-needle aspiration [207]. Benign cysts are rubber-like in texture and move about within the glandular breast tissue, chest wall, and skin. Except for inflammatory cysts, a patient's discomfort and tenderness are either absent or mild [216]. Upon additional clinical and diagnostic investigation, the majority of patients present with multiple cysts. These cysts are seen as well-circumscribed, oval to circular, anechoic, or hypoechoic foci of varied sizes on ultrasonography. On the other hand, simple cysts must be distinguished on ultrasonography from complex cysts. Only large lesions that induce persistent symptoms require aspiration. The color and viscosity of cyst fluid can range from a clear, thin content to a whitish, opaque secretion to a dirty-green, bluish, or gray secretion. Notwithstanding, the color has no diagnostic value [204].

### 11.3 Risk factors of FBD

Gallicchio et al. showed weakly relationship between breast cancer and ESR1 variations and one of the four ESR2 variants investigated in 1438 Caucasian women with benign breast disease [217]. Obesity and excess body fat, according to current knowledge, are the causes of increased estradiol and estriol production (product of estradiol transformation) suggesting that a higher BMI may have an indirect effect on the development of FBD (through hyperestrogenemia) [214].

## 12 Ductal pathology

### 12.1 Ductal hyperplasia

The terminal duct lobular unit (TDLU) is formed when normal breast ducts come to an end. The duct terminates in lobules made up of acini, which are tiny glandular structures. Inner luminal epithelial cells and outer luminal myoepithelial cells make up the bilayer that lines the ductal-lobular systems [218]. The TDLU is where the majority of breast lesions (both benign and malignant) occur. Atypical ductal hyperplasia (ADH) is clonal epithelial cell proliferation within the duct. The TDLU or the interlobular ducts are involved in ADH. ADH has small, spherical, monomorphic, non-overlapping cells with homogeneous nuclei, uncommon mitosis, and inconspicuous nucleoli, as well as other atypical histological characteristics [219].

In breast tissue, atypical ductal hyperplasia is a pathogenic condition. Atypical Ductal Hyperplasia (ADH) is generally discovered by chance on needle biopsy specimens taken in response to aberrant mammography findings. Because atypical ductal hyperplasia is associated with an increased risk of breast cancer, it is classified as a "high-risk" lesion rather than a "precursor" lesion. The difference is that breast cancer associated with ADH can occur anywhere in the breasts, not just in the ADH area. The true incidence of ADH is unknown because most cases are discovered by chance. Once discovered, it is known to raise the risk of breast cancer by about fivefold [220]. It is critical to address risk reduction techniques if atypical ductal hyperplasia has been diagnosed and breast cancer has been ruled out. Moreover, the use of tamoxifen as a treatment option for these women is one such measure [221]. If a core needle biopsy reveals breast cancer, a more comprehensive excisional biopsy is required to rule out the disease. If on excisional biopsy, only ADH is discovered, the patient is surgically complete. This includes cases with positive margins. There is no need for node sampling or mastectomy because ADH is not a malignancy [222].

#### 12.1.1 Role of estrogen in ADH

Estrogen is thought to play a role in the pathophysiology of breast cancer by promoting normal growth of the breast epithelium through the estrogen receptor's mechanism of absorption into the cell [223]. As mentioned earlier, ER expression is found in both normal and malignant breast epithelium to a greater extent [224]. In both normal breast epithelium and ductal hyperplasia of the usual type, ER expression appears to increase with age, considering ER expression is generally low in the normal ductal epithelium and higher in proliferative breast disease, particularly when linked with atypia and carcinoma in situ [225]. Increased ER expression in normal ductal hyperplasia has also been linked to breast cancer risk in one case-control study [226]. However, no studies have looked at the impact of ER expression on breast cancer risk in women with atypical hyperplasia, which is a known higher-risk group [227]. Moreover, long-term follow-up of the NSABP P-1 study, one of the largest prospective breast cancer prevention trials, has revealed a risk decrease of breast cancer in women with past atypical hyperplasia taking tamoxifen, presumably through the ER [221].

These clinical findings, along with previous research on ER expression in other benign breast epitheliums, suggest that ER expression in atypical hyperplasia could be a predictor of breast cancer risk in the future. The researchers wanted to look at ER expression in atypical hyperplasia and see if there were any links to age at biopsy, the reason for biopsy, type of atypia, number of atypical foci, involution status, and family history, as well as see if there was any link between ER expression in atypia and subsequent breast cancer risk [221]. A large cohort of women with atypical hyperplasia was studied, and it was discovered that ER expression is higher in atypical ductal hyperplasia than in atypical lobular hyperplasia. In contrast to previous research, they discovered that ER expression increased with age at the time of diagnosis of atypical hyperplasia. Despite evidence that estrogen exposure is linked to an increased risk of breast cancer and that atypical hyperplasia has more ER expression than normal breast epithelium, the degree of ER expression in atypical hyperplasia does not correlate with the risk of breast cancer [228].

### 12.2 Carcinoma In Situ (CIS)

Carcinoma In Situ (CIS) of the breast is a heterogeneous collection of lesions that covers a broad range of clinical and histological changes. Biologically; CIS can range from biologically aggressive lesions with a high chance of progressing to invasive carcinoma to lesions with very low malignant potential. There are two forms of CIS, ductal carcinoma in situ (DCIS) and lobular carcinoma in situ (LCIS). CIS is common, and it is expected that roughly 20% to almost a third of all women will get it during their lives [229].

#### 12.2.1 Ductal Carcinoma In Situ (DCIS)

DCIS is an early, localized stage of breast cancer [230]. It accounts for up to 15% of newly diagnosed breast tumors, and microcalcifications are usually used to detect them [231]. Moreover, a considerable majority of these tumors will progress to invasive carcinoma if left untreated. DCIS, on the other hand, has a favorable prognosis when properly treated [230]. In individuals with non-palpable, mammographically discovered DCIS, mammographically guided wire biopsy remains the gold standard for achieving a histological diagnosis [232]. The optimal management of DCIS remains controversial. The goal of DCIS treatment is to keep the disease localized and prevent it from progressing to invasive carcinoma [230]. Mastectomy, local excision with radiation therapy, and local excision alone are all possible treatment choices [232]. Total mastectomy was the treatment of choice for DCIS for decades, and it should still be considered the gold standard against which more conservative treatments must be measured. Additionally, mastectomy is related to a 1% chance of chest wall recurrence, and axillary lymph node dissection is not routinely recommended. On the other hand, mastectomy is likely overtreatment in a significant number of patients, particularly those with minor, mammographically diagnosed lesions. Moreover, local excision alone has been recommended in carefully selected individuals, whereas the rest of the patients undergoing breast-conservation surgery should get breast irradiation. There is evidence that breast-conservation therapy is an effective alternative in the management of selected DCIS patients. The use of radiotherapy after lumpectomy reduces the risk of recurrence significantly. The most common predictors of recurrence include nuclear grade, comedo necrosis, and margin involvement. Adjuvant chemotherapy has no role in the treatment of this disease. Although tamoxifen's significance in the treatment of DCIS is unclear, it should only be used in patients who are participating in clinical trials. Approximately half of all tumors relapse as invasive cancer after breast conservation therapy. The majority of patients with recurrent disease can be properly treated, usually with a salvage mastectomy, but in certain cases with breast-conservation therapy [231].

#### 12.2.2 Lobular Carcinoma In Situ (LCIS)

This form of breast cancer starts in lobules and terminal ducts and is more common and more frequent than this incident would seem to suggest. When the tumor invades, it often does so in an odd way that enables one to recognize the likelihood of such an origin even though it is hard to trace it after some experience. Furthermore, it is frequently possible to find peripheral areas where lobular carcinomatosis in situ is still clearly visible in the completely infiltrative form. Thus, out of these same 300 examples, 5 had a very distinct pattern, 2 had one that was only moderately developed, and 5 had one that was certainly there but was insufficient [233].

### 12.3 Invasive ductal carcinoma (IDC)

Invasive ductal carcinoma (IDC) is a highly malignant subtype of breast cancer that belongs to the classification of epithelial tumors. It is the general designation for nonspecific invasive carcinoma that belongs to the epithelial tumors classification group. IDC is the most frequent kind of breast cancer in women, and it is also the main cause of cancer-related death [234]. The most dramatic transcriptome alteration occurs at the normal to DCIS transition, while others have found that different stages of breast cancer (ADH, DCIS, and IDC) are highly similar at the transcriptome level [235].

## 13 Estrogen-secreting tumors in developing breast cancer

Granulosa cell tumors (GCT) and thecoma are called estrogen-producing tumors. Researchers found that there is a relationship between these tumors and an increased risk of breast cancer [236]. With the Danish female population as a benchmark, the team of Hammer Anne discovered evidence of a markedly higher incidence of breast cancer in women with GCT. Only a few papers on the likelihood of breast cancer in women with GCT were found by them. Ohel et al. discovered a 6.4% incidence of breast cancer in 172 women during a 15-year follow-up study carried out in 1983, which is similar to the data of Hammer Anne et al. study [[236], [237], [238], [239]]. 200 women with GCT or theca cell tumors had a 5.5% incidence of breast cancer, according to Evans et al. study. Three women were diagnosed with breast cancer before the GCT diagnosis, and eight other women received the diagnosis. In the study by Björkholm et al., 936 women who had been diagnosed with either a GCT or a theca cell tumor had a 2% incidence of breast cancer. Four women received diagnoses concurrent with GCT diagnoses, while 15 women received diagnoses post-GCT diagnoses. In addition, reports of an increased incidence of thyroid cancer, endometrial carcinoma, lymphoma, and colon cancer were made [237,238]. In these investigations, there was no distinction made regarding the incidence of breast cancer between women with GCT and those with theca cells. Therefore, it is unknown what the actual incidence of breast cancer is among women with GCT. However, Björkholm reported a 3.3% incidence of breast cancer among 153 GCT women in different research. Björkholm also discovered that GCT women had a considerably higher risk of cardiovascular disease in this investigation. Although the hormonal balance being disturbed was proposed as a potential cause, this has not been supported by other research [239].

## 14 Role of estrogen in breast cancer in males

Male breast cancer is a rare type of cancer that accounts for fewer than 1% of all malignancies in men and 1% of all breast cancers. However, the incidence of the disease is increasing, reaching 15% in some patient groups throughout their lives [240]. It is most common in people over the age of 60 ^241^. There are already identifiable risk factors such as constitutional, environmental, hormonal (abnormalities in estrogen/androgen balance), and genetic (positive family history, Klinefelter syndrome, mutations in BRCA1 and especially BRCA2) factors. In 75% of cases, the clinical manifestation is a painless hard and fixed nodule in the subareolar region, with nipple commitment occurring earlier than in women [242]. The most common type of male breast cancer is infiltrating ductal carcinoma which represents around 70–90%. Only 7% of cases of in situ but non-invasive cancer are ductal [241]. Clinical examination, combined with a confirming biopsy, is still the most important step in evaluating men with breast lesions [242]. The conventional basic treatment is Patey's mastectomy, which is a modified radical mastectomy with the removal of certain lymph nodes [241]. Hormone metabolic abnormalities linked to high estrogen or prolactin levels appear to be the causative mechanism. Progesterone and estrogen receptors are found in tumors in men more commonly than in women. According to immunohistochemistry and the vast majority of cases are estrogen receptor alpha (ER) positive. This indicates that this steroid hormone receptor plays a role in tumor formation and progression [243]. Also, about 75% of all tumors express estrogen and progesterone receptors, which become more positive as the patient gets older [244].

## 15 Conclusion

Estrogen homeostasis and tissue-specific exposure to estrogen and its metabolites are influenced by genetic and environmental variables. Uncertainty exists regarding the relative impact of the changing serum estrogen levels linked to the menstrual cycle in premenopausal women and the more constant levels in postmenopausal women on the total lifetime exposure to estrogen. When considered collectively, the body of evidence is consistent with the idea that estrogen and its metabolites are connected to both the beginning and advancement of breast cancer, albeit these connections are complicated. Recent findings from sizable clinical trials of selective estrogen receptor modulators provide more proof of the link between estrogen and the risk of breast cancer. Tamoxifen's antiestrogenic impact lowered the incidence of breast cancer in healthy premenopausal and postmenopausal women who were at higher risk for the condition, while raloxifene did the same for postmenopausal women who had osteoporosis. Although a link between estrogen exposure and the risk of breast cancer has been shown in some populations of women, the risk for a given woman cannot be precisely predicted. Breast density on mammography, serum estrogen concentrations, and bone mineral density are a few clinical indicators of estrogen exposure that may help determine a woman's risk of breast cancer. Composite risk assessments based on these and other risk variables, such as family and reproductive histories, may help us better understand the role of estrogen in the pathogenesis of breast cancer and provide a more accurate evaluation of risk for specific women.

## Author contribution

Conceptualization: MSA and RMA. Study design, data collection, and data analysis: KA, SA and AK. Writing-Original draft preparation: KA, AK, SA, AMA, SAS, RAB, SFA, AMA, and SHB. Writing- Reviewing and Editing the final manuscript: RMZ and MSA. All authors reviewed, contributed, and approved the final manuscript version.

## Funding

None.

## Implications and contribution

Many of the factors highlighted in the review should be considered by researchers studying the impact of estrogens on breast cancer disease (BCa).

## Data availability statement

Data will be made available on request.

## Declaration of competing interest

The authors declare that they have no known competing financial interests or personal relationships that could have appeared to influence the work reported in this paper.
